# 

**Published:** 1916-12

**Authors:** 


### CONTENTS.

Page

The Art of Medicine in the Age of Homer. By F. H. Edgeworth, M.D., D.Sc., M.A. ... 105

The Effect of General Conditions on the Quality of the Teeth. By Major W. R. Ackland, R.A.M.C. (T.). ... 118

Some Aspects of British Surgery in France. By James Swain, M.S., M.D. Lond., F.R.C.S. ... 128

Medicine and Surgery in Mesopotamia. By Captain Carey F. Coombs, R.A.M.C. (T.). ... 136

Ten Months in France with a Field-Service X-Ray Outfit. By William Cotton, Capt., R.A.M.C. (T.). ... 144

Notes on the Operation for Drainage of the Sphenoidal Sinus. By P. Watson-Williams, Major, R.A.M.C. (T.). ... 157

Progress of the Medical Sciences:

Dermatology ... 178

Reviews of Books ... 180

Editorial Notes ... 186

The Library of the Bristol Medico-Chirurgical Society ... 195

Obituary Notice ... 201

Local Medical Notes ... 202

